# Supplementary material for: Topological electronic structure and spin texture of quasi-one-dimensional higher-order topological insulator Bi4Br4
Source: Nat Commun. 2023 Dec 7;14:8089. doi: 10.1038/s41467-023-43882-z (PMC10703900; doi:10.1038/s41467-023-43882-z)
Supplement: Supplementary file 1 — Supplementary Information [file 41467_2023_43882_MOESM1_ESM.pdf]

Supplementary Information for

Topological electronic structure and spin texture of quasi-one-dimensional higher-order topological insulator Bi_4Br_4

Wenxuan Zhao[1], Ming Yang[2,3], Runzhe Xu[1], Xian Du[1], Yidian Li[1], Kaiyi Zhai[1], Cheng Peng[4], Ding Pei[4], Han Gao[5], Yiwei Li[5], Lixuan Xu[1], Junfeng Han[6,7], Yuan Huang[6,7], Zhongkai Liu[5,8], Yugui Yao[6,7], Jincheng Zhuang[2,3], Yi Du[2,3*], Jinjian Zhou[6*], Yulin Chen[4,5,8*], and Lexian Yang[1,9,10*]

[1]*State Key Laboratory of Low Dimensional Quantum Physics, Department of Physics, Tsinghua University, Beijing 100084, China.*

[2]*School of Physics, Beihang University, Beijing 100191, China.*

[3]*Centre of Quantum and Matter Sciences, International Research Institute for Multidisciplinary Science, Beihang University, Beijing 100191, China.*

[4]*Department of Physics, Clarendon Laboratory, University of Oxford, Parks Road, Oxford OX1 3PU, UK.*

[5]*School of Physical Science and Technology, ShanghaiTech University and CAS-Shanghai Science Research Center, Shanghai 201210, China.*

[6]*Centre for Quantum Physics, Key Laboratory of Advanced Optoelectronic Quantum Architecture and Measurement (MOE), School of Physics, Beijing Institute of Technology, Beijing 100081, China;*

[7]*Yangtze Delta Region Academy of Beijing Institute of Technology, Jiaxing 314001, Zhejiang province, China*

[8]*ShanghaiTech Laboratory for Topological Physics, Shanghai 200031, China.*

[9]*Frontier Science Center for Quantum Information, Beijing 100084, China.*

[10]*Collaborative Innovation Center of Quantum Matter, Beijing, China.*

[*]e-mail: DY: yi_du@buaa.edu.cn; JJZ: jjzhou@bit.edu.cn; YLC: yulin.chen@physics.ox.ac.uk;

LXY: lxyang@tsinghua.edu.cn.

This file includes the following contents:

Supplementary Note 1: Characterization of Bi_4Br_4 samples;

Supplementary Note 2: ARPES spectra measured at 30 K;

Supplementary Note 3: Comparison between laser-based μ-ARPES measurements on the (001) and (100) surfaces;

Supplementary Note 4: Visualization of the edge states using scanning tunneling spectroscopy.

Supplementary Note 5: Calculated spin polarization of the (100) surface states;

Supplementary Note 6: Calculated spin polarization of the hinge states;

Supplementary Note 7: Polarization-dependent spin-ARPES measurements on the (100) surface;

Supplementary Note 8: Temperature dependence of ARPES spectra;

Supplementary Note 9: ARPES spectra upon alkali doping;

Supplementary Figure 1-9.

Supplementary Notes

1. Characterization of Bi_4Br_4 samples.

The Bi_4Br_4 samples used in ARPES experiments were characterized by different methods as shown in Supplementary Fig. 1. The single crystalline X-ray diffraction (XRD) measurement is in good agreement with the previous data and suggests the high quality of our samples (Supplementary Fig. 1a). The sharp Bi and Br core-level peaks in X-ray photoemission spectroscopy (XPS) data confirm the sample composition (Supplementary Fig. 1b). Supplementary Fig. 1c shows the temperature dependence of the electronic resistance of Bi_4Br_4 along the b axis, which is in good agreement with previous studies. With decreasing temperature, the resistivity first decreases until about 75 K, then quickly increases. At low temperatures, the resistance increases at a rate slower than the exponential law, which is attributed to the metallic edge state or a Lifshitz transition[1].

2. ARPES spectra measured at 30 K.

Supplementary Fig. 2a shows the ARPES spectra on the (100) surface of Bi_4Br_4 detected at 30 K. The surface valence/conduction bands and the surface gap of about 40 meV can be clearly resolved in the curvature plot of the spectra (Supplementary Fig. 2b), similar to the results in the main text. To visualize the fine structure inside the surface gap, the data were deconvoluted to remove the thermal broadening and experimental resolution effects [Fig. 2c], which is a commonly-used method in ARPES data analysis[2]. We observe dispersive features inside the (100) surface gap. In particular, the in-gap states show a double-crossing dispersion that nicely agrees with the calculated hinge states (Supplementary Fig. 2d). We therefore argue that the dispersive states inside the (100) surface gap are contributed by the hinge states of Bi_4Br_4.

3. Comparison between laser-based μ-ARPES measurements on the (001) and (100) surfaces.

Supplementary Fig. 3 compares ARPES data measured on the (001) and (100) surfaces using laser-based μ-ARPES. The data on the (100) surface has been shown in the main text in which we observe the gapped surface states and in-gap states. On the (001) surface, by contrast, we observe the bulk valence band with the band top at about E_F - 0.25 eV, consistent with the results by synchrotron-based ARPES in Fig. 2b of the main text. No surface state is observed on the (001) surface, consistent with the *ab-initio* calculation in Fig. 1g.

Interestingly, we notice weak but resolvable spectral weight near E_F in Supplementary Fig. 3b. Supplementary Fig. 3c compares the energy distribution curves at k_y = 0 collected on the (001) and (100) surfaces. The EDC of the (001) surface shows considerable spectral weight (manifested as a shoulder) at the energy position corresponding to the (100) surface gap. These states cannot be induced by the mixed signal from the (100) domains since the more intensive conduction and valence bands of the (100) surface states were not observed. Moreover, our micro-ARPES with sub-micron spatial resolution excludes the mixture of the (100) domains on the (001) cleavage surface (no conduction and valence (100) surface states have been observed on the (001) cleaved samples). Since the (001) and (100) surfaces share the hinges, the extra states observed on the (001) surface are naturally attributed to the edge states accommodated by the terraces after sample cleavage and thus provide evidence for the HOTI phase of Bi_4Br_4.

4. Visualization of the edge states using scanning tunneling spectroscopy (STS).

The detection of edge states can be directly performed by scanning tunneling microscopy, supporting the non-trivial topological phase of Bi_4Br_4. We perform STS measurements on the (001) surface of Bi_4Br_4 (unfortunately, the STS experiment on the (100) surface is not possible at present). Supplementary Fig. 4a shows the topography near the edges on the (001) surface. In the STS map, we clearly observe enhanced

density of states at both edges of the terrace near the Fermi level, which directly visualizes the in-gap edge states. The spatial width of the edge states is several nanometers, which agrees with the previous works[1,3].

5. Calculated spin polarization of the (100) surface states.

Supplementary Fig. 5 shows the calculated spin texture of the band dispersion of the (100) surface states along the chain direction (k_y). The conduction and valence surface bands exhibit opposite z'- and x'-component of spin polarization. The z'-component of the spin polarization of the conduction band reaches nearly 100%, while the x'-component is about 50% and the y-component is nearly zero, which are qualitatively consistent with our spin-ARPES measurements in Fig. 4 of the main text.

6. Calculated spin polarization of the hinge states.

The spin texture of the (100) surface states originates from the helical spin structure of the quantum-spin Hall edge state of monolayer Bi_4Br_4. Similarly, the hinge states also exhibit helical spin texture. Supplementary Fig. 6 shows the spin polarization of the hinge states calculated on a 10-layer ribbon with a width of 30 Bi_4Br_4 chains. At the same momentum positions, the two hinge modes exhibit opposite z'- and x'-component of spin polarization. The z'-component of the spin polarization of the hinge states reaches nearly 100%, compared to about 50% of the x'-component. Similar to the (100) surface states, the y-component of the polarization of the hinge states is nearly zero. However, due to the weak intensity of the hinge states compared to the (100) surface states, the mixture of signals from different hinges, and limited resolutions of spin-ARPES, no spin polarization of the hinge states can be detected.

7. Polarization-dependent spin-ARPES measurements on the (100) surface.

The spin polarization of the boundary states of quantum materials may depend on the polarization of the excitation light[4]. The schematic illustration in Supplementary Fig. 7a shows the ARPES measurements on the (100) surface using linear-horizontally (LH) and linear-vertically (LV) polarized photons. The measured z'-component of the spin polarization shows no dependence on the laser polarization, suggesting that the spin-ARPES measurement reflects the intrinsic spin texture of the (100) surface states of Bi_4Br_4.

8. Temperature dependence of ARPES spectra.

We perform laser-based ARPES measurements on the (100) surface of Bi_4Br_4 at different temperatures (from 80 K to 295 K) as shown in supplementary Fig. 8. The band dispersion of the gapped surface states does not exhibit noticeable changes upon increasing temperature. By contrast, the bulk valence band shifts towards the Fermi level with increasing temperature. This shift of the bulk Fermi surface has been observed and attributed to the defect effects. It is noteworthy that previous temperature-dependent measurements suggested a temperature-induced Lifshitz transition[1], which is absent in our experiment.

9. ARPES spectra upon alkali doping.

To explore possible tunability of the (100) surface gap and the hinge states, we perform surface doping of Rb atoms in Supplementary Fig. 9. The band dispersion of the (100) surface states shows little changes upon slight Rb doping, except for a chemical potential shift. With further Rb doping, the ARPES spectra get blurred and the bulk conduction band is observed after surface doping for 205 s. The bulk gap is estimated to be about 300 meV.

Supplementary Figures

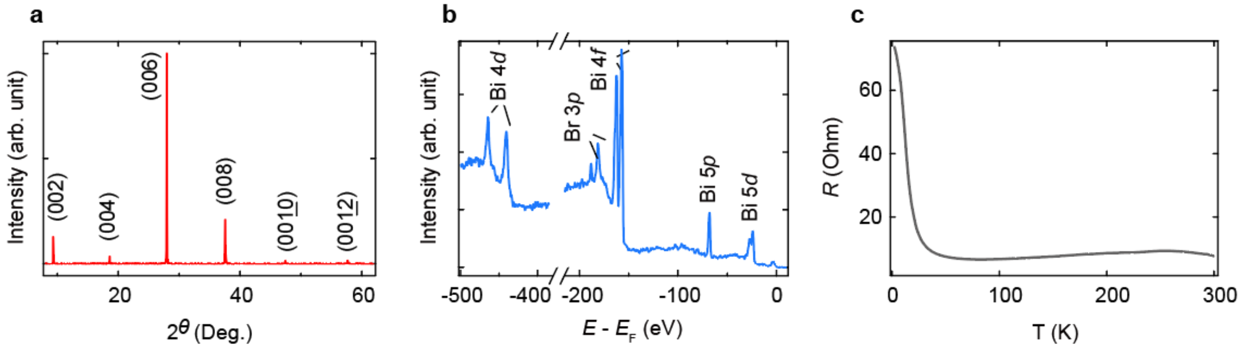

Supplementary Fig.1 | Characterization of Bi_4Br_4 samples. a, X-ray diffraction pattern measured along [001]. **b**, Core-level peaks measured with X-ray photoemission spectroscopy (XPS). **c**, Resistance as a function of temperature.

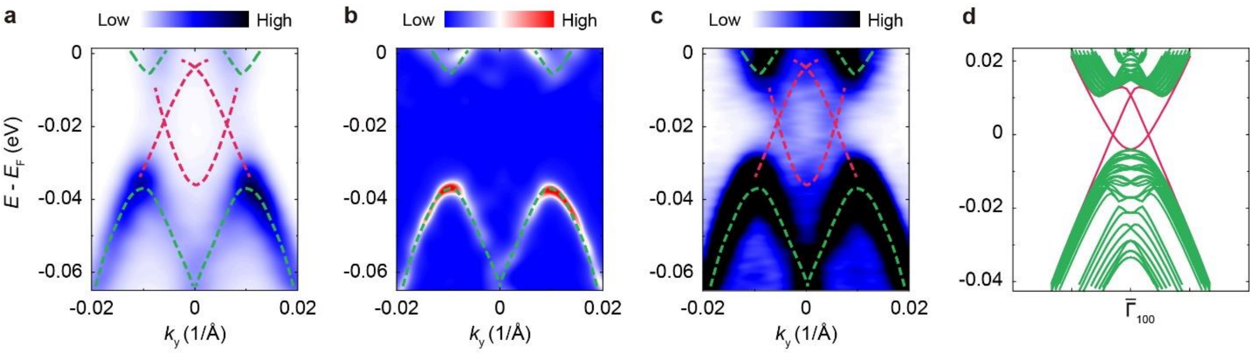

Supplementary Fig.2 | ARPES spectra measured at 30 K. a, ARPES spectra detected at 30 K. **b**, Curvature plot of the spectra in **a** showing the (100) surface band gap. **c**, ARPES spectra after deconvolution to remove the spectral broadening effect. The green and red dashed lines in **a**-**c** are guide to eyes indicating the dispersion of the (100) surface states and hinge states. **d**, *Ab-initio* calculation of the electronic structures of a 10-layer Bi_4Br_4 slab (the same as Fig. 3a in the main text).

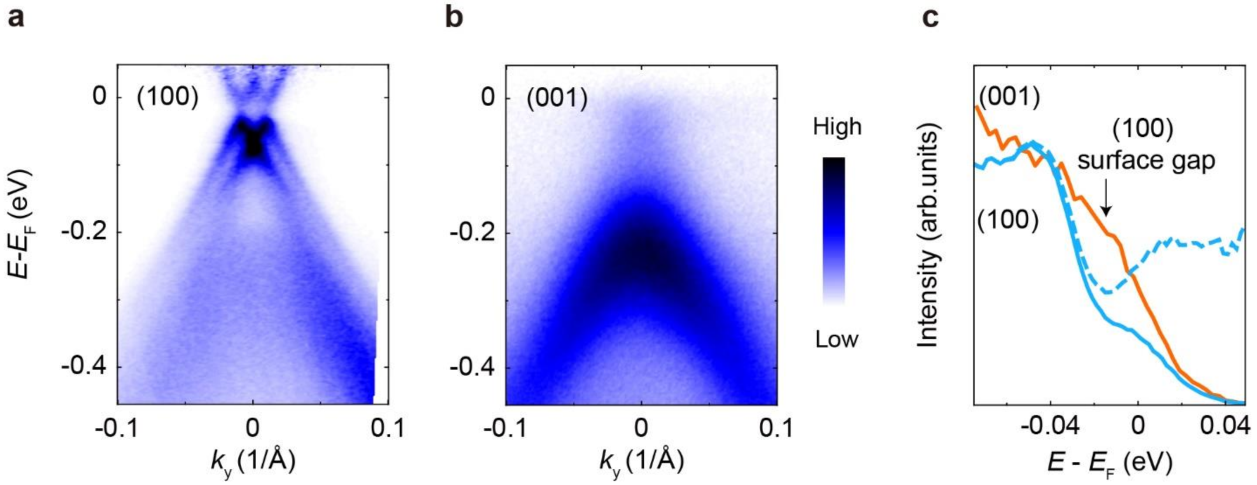

Supplementary Fig.3 | Comparison between laser-based μ-ARPES measurements on the (001) and (100) surfaces. **a**, **b**, ARPES spectra along the chain direction measured on the (100) and (001) surfaces of Bi_4Br_4, respectively. **c**, Comparison between the energy distribution curves (EDCs) at $k_y = 0$ (integrated with a momentum range of 0.03 1/Å) measured on the (100) (blue line) and (001) (orange line) surfaces. The blue dashed line is the (100) EDC divided by the Fermi-Dirac function.

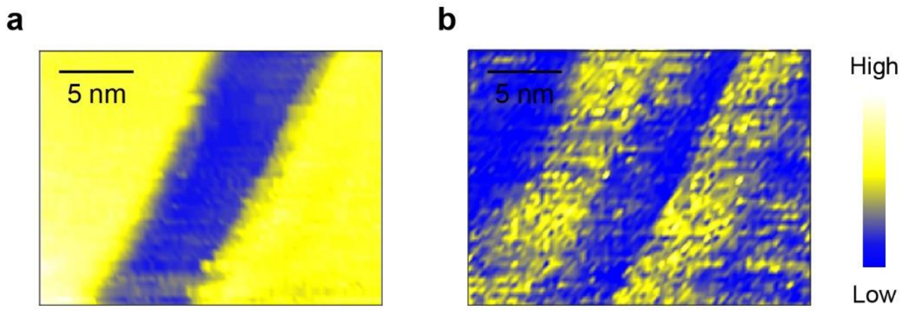

Supplementary Fig.4 | Visualization of the edge states using scanning tunneling spectroscopy. **a**, Topography of the (001) surface edge of Bi_4Br_4. **b**, The dI/dV map detected at the Fermi energy with the same range of **a**.

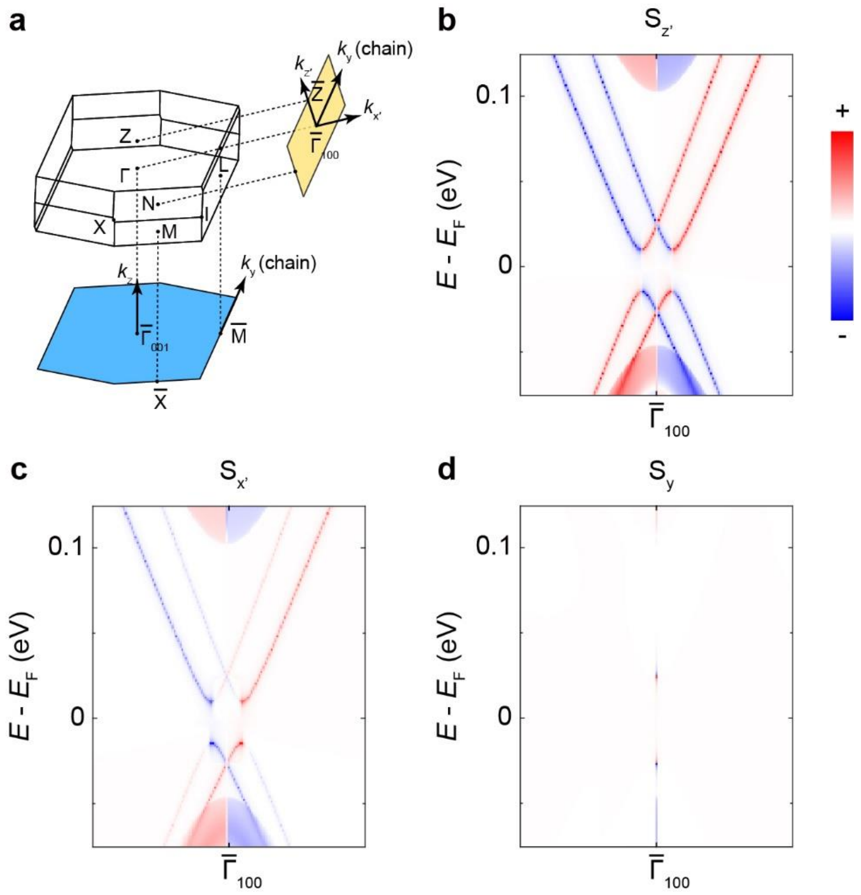

Supplementary Fig.5 | Calculated spin polarization of the (100) surface states. a, Bulk and surface Brillouin zone with high-symmetry points and directions indicated. **b-d**, Calculated *z′*-, *x′*- and *y*-component of the spin polarization, respectively.

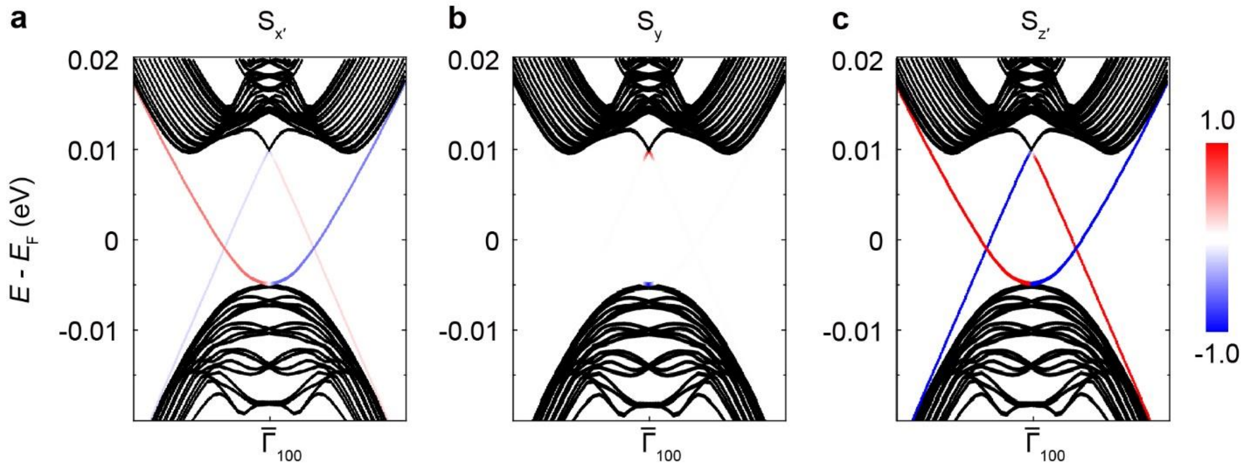

Supplementary Fig.6 | Calculated spin polarization of the hinge states. a-c, *x′*-, *y*-, and *z′*-component of the spin polarization of the hinge states.

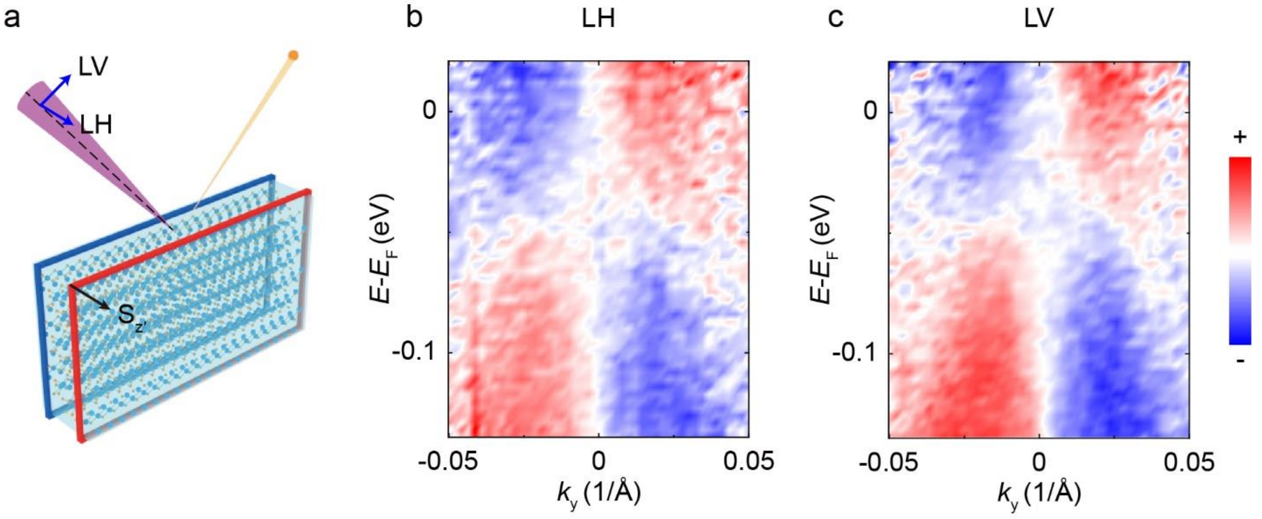

Supplementary Fig.7 | Polarization-dependent spin-ARPES spectra of Bi_4Br_4 measured on the (100) surface. **a**, Schematic of photoemission measurement on the (100) surface of Bi_4Br_4. The black and blue arrows indicate the $S_{z'}$ direction and photon polarizations respectively. **b**, **c**, The z'-component of spin-polarized ARPES spectra using linear-horizontally (vertically) polarized laser beams.

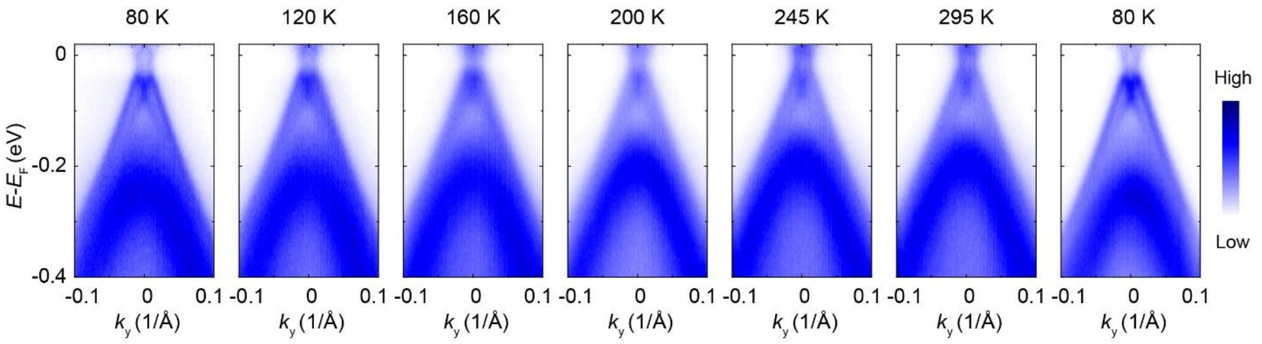

Supplementary Fig.8 | Temperature dependence of ARPES spectra. The temperature-dependent ARPES measurements are performed on the (100) cleavage surface of Bi_4Br_4 using a 7-eV laser beam. The spectra are divided by the Fermi-Dirac function.

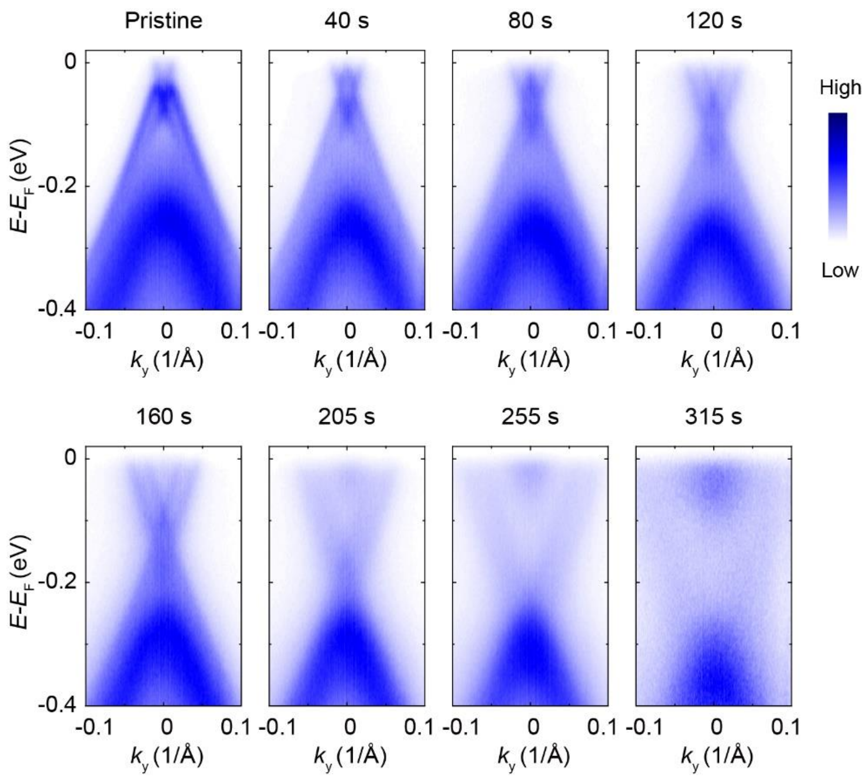

Supplementary Fig.9 | ARPES spectra upon alkali doping. The ARPES spectra are measured on the (100) cleavage surface of Bi_4Br_4 upon Rb surface doping.

Supplementary References:

1 Yang, M. *et al.* Large-gap quantum spin Hall state and temperature-induced Lifshitz transition in Bi_4Br_4. *ACS Nano* **16**, 3036-3044 (2022).

2 Razzoli, E. *et al.* Evolution from a Nodeless Gap to d_{x2-y2}-Wave in Underdoped $La_{2-x}Sr_xCuO_4$. *Phys. Rev. Lett.* **110**, 047004 (2013).

3 Shumiya, N. *et al.* Evidence of a room-temperature quantum spin Hall edge state in a higher-order topological insulator. *Nat. Mater.* **21**, 1111-1115 (2022).

4 Jozwiak, C. *et al.* Photoelectron spin-flipping and texture manipulation in a topological insulator. *Nat. Phys.* **9**, 293-298 (2013).
